# Circulating biomarkers in renal cell carcinoma: the link between microRNAs and extracellular vesicles, where are we now?

**DOI:** 10.15586/jkcvhl.2014.19

**Published:** 2014-12-24

**Authors:** Ana L Teixeira, Francisca Dias, Mónica Gomes, Mara Fernandes, Rui Medeiros

**Affiliations:** 1 Molecular Oncology and Viral Pathology Group, Portuguese Institute of Oncology of Porto, Porto, Portugal; 2 Research Department, Portuguese League Against Cancer (NRNorte), Porto, Portugal; 3 Abel Salazar Institute for the Biomedical Sciences (ICBAS) of University of Porto, Porto, Portugal; 4 Faculty of Health Sciences of Fernando Pessoa University, Porto, Portugal

## Abstract

Renal cell carcinoma (RCC) is a lethal urological cancer, with incidence and mortality rates increasing by 2-3% per decade. The lack of standard screening tests contributes to the fact that one-third of patients are diagnosed with locally invasive or metastatic disease. Moreover, 20-40% of RCC patients submitted to surgical nephrectomy will develop metastasis. MicroRNAs (miRNAs) are small non-coding RNAs responsible for gene regulation at a post-transcriptional level. It is accepted that they are deregulated in cancer and can influence tumor development. Thus, miRNAs are promising RCC biomarkers, since they can be detected using non-invasive methods. They are highly stable and easier to quantify in circulating biofluids. The elevated miRNA stability in circulating samples may be the consequence of their capacity to circulate inside of extracellular microvesicles (EMVs), for example, the exosomes. The EMVs are bilayered membrane vesicles secreted by all cell types. They can be released in the interstitial space or into circulating biofluids, which allows the travelling, binding and entrance of these vesicles in receptor cells. This type of cell communication can shuttle bioactive molecules between cells, allowing the horizontal transference of genetic material. In this review, we focus on circulating miRNAs (miR-210, miR-1233, miR-221, miR-15a, miR-451, miR-508, miR-378) in the biofluids of RCC patients and attempt to establish the diagnostic and prognostic accuracy, their synergic effects, and the pathways involved in RCC biology.

## Introduction

Renal cell carcinoma (RCC) is thought to arise from the renal parenchyma and it is the most common solid tumor in the adult kidney, accounting for 2-3% of all cancers (1). Worldwide mortality from RCC exceeds 100,000 patients each year with the incidence and mortality rate increasing by 2–3% per decade (1–3). RCC is the most lethal common urological cancer, with a cancer-specific mortality of 30–40%, compared to 20% mortality rates for bladder and prostate cancers (1). The high RCC incidence rate could be partially explained by the improvement of the diagnostic tests (computed tomography, MRI and so on) which allows the detection of a significant number of incidental and asymptomatic cases (2). However, there is no standard screening tests for RCC, and one third of patients present with metastatic RCC (mRCC) at the time of diagnosis. Moreover, over the course of the disease, 20-30% of patients treated with surgery will relapse (4).

The RCC frequency in men is 1.5-2.0 times greater than in woman, with an age peak around 60-70 years (5). The exact RCC etiology remains unclear, although lifestyle risk factors such as cigarette smoking and obesity, and iatrogenic factors like hypertension, use of antihypertensive medications and acquired renal cystic disease have been identified as potential risk factors (6, 7).

According to the World Health Organization there are three major RCC histological subtypes in adults: the clear cell RCC (ccRCC) that occurs in 75-80% of cases, the papillary RCC (10-15%) and the chromophobe RCC (4-5%) (1). These histologic subtypes reflect the tumor heterogeneity and the occurrence of distinct molecular alterations during the course of the disease.

Surgical intervention is the primary approach for the treatment of RCC detected at early stage. However, surgery alone has a limited benefit in patients with metastatic disease, except for palliative reasons (3, 8). Until the past decade, the treatment options for patients with mRCC have been extremely limited, as RCC is notoriously resistant to cytotoxic chemotherapy and radiotherapy (9, 10). Prior to the use of antiangiogenic agents, systemic treatment options for mRCC were limited to cytokine therapies interleukin-2 (IL-2) and interferon-alpha (IFN-α), but they were proved to be ineffective since only a small percentage of the patients showed benefit in terms of long term disease-free survival (11, 12). Currently, targeted therapies have become the standard of care for patients with mRCC with significant impact in patient outcome, replacing the cytokine therapy (13).

The targeted therapies include receptor tyrosine kinase inhibitors (TKIs), vascular endothelial growth factor (VEGF) antibodies, and mammalian target of rapamycin inhibitors (mTORs) (3, 14, 15). Although the outcome of patients has improved, many tumors develop resistance to targeted therapies due to compensatory changes within the target pathway that bypass the site of inhibition (13, 16). Usually, resistance to the targeted agents has been shown to develop after a median of 5–11 months of treatment and a small subset of patients do not experience any clinical benefit from the targeted therapy (13).

No standard approaches to biomarker sampling or analysis have been adopted for RCC since many of the putative tumor markers themselves are still under active investigation for further validation (17). The ideal biomarker must be accessible using non-invasive protocols, inexpensive to quantity, specific to the disease of interest, a reliable early indicator of disease before clinical symptoms appear and a way to stratify the disease and assess response to therapy (18). Despite being one of the most rapidly growing areas in cancer research, the establishment of biomarkers in body fluids has not been an easy task (19). One of the major challenges that need to be overcome is the susceptibility to degradation of the circulating biomarkers by proteases and nucleases. On the other hand, there is also the problem of the endogenous production of biomarker molecules by normal cells that may artificially augment the biomarker signals (20, 21).

Plasma and serum have been the focus of extensive research for the past years (22). However, serum and plasma-based tests suitable for clinical use in early tumor detection are currently limited (23, 24). Nowadays, the majority of the routinely used serum markers are proteins and the standard methodologies used to measure them remain labor-intensive (23). The same is true for urine samples. Urine metabolomics analysis is theoretically promising but difficulties with the heterogeneous nature of urine metabolomics, potential contamination of non-human metabolites from genitourinary flora, and special handling requirements have limited the progress of its use as a source of biomarkers for RCC (25).

Of the possible non-invasive biomarkers that have been studied in RCC, the ones that seem more promising are the miRNAs, since they can be detected using non-invasive methods and are easier to quantify when compared to proteins. However, further research is needed in order to validate them (26–28).

## MicroRNAs

**Figure 1.**
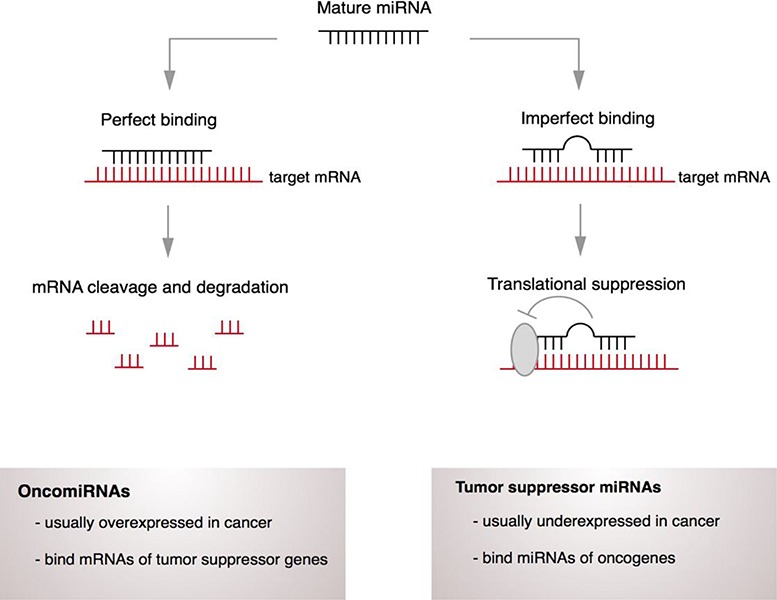
Mechanism of miRNA action. MiRNA can bind to specific regions of target mRNA transcripts and destabilizes the target transcript and/or blocks its translation.

MiRNAs are a class of small non-coding RNAs (19-25 nucleotides in length), that are involved in the regulation of biological processes, including cell proliferation and differentiation. miRNAs regulate gene expression by sequence-selective targeting of mRNAs, leading to their degradation or blockade at the post-transcriptional level, depending on the degree of complementarity between miRNAs and the target mRNA sequence (Figure 1) (29–32). They arise from intergenic or intragenic genomic regions that are transcribed as long primary transcripts. Then, the primary transcripts undergo processing steps that involve Drosha and Dicer enzymes, to form a mature miRNA. The mature miRNA binds to specific regions of target mRNA transcripts and destabilizes the target transcript or blocks its translation (33, 34). MiRNA expression is dynamic, since it is postulated that each miRNA regulates up to 100 different mRNAs and that more than 10000 mRNAs appear to be directly regulated by miRNAs (35). Many miRNAs have been identified to act as oncogenes, tumor suppressors or even modulators of cancer stem cells and metastasis formation (36). OncomiRs are known to down-regulate tumor suppressor genes, and have been reported to be overexpressed in multiple miRNA-profiling studies. On the other hand, tumor suppressor miRNAs are responsible for down-regulating oncogenes, and are mostly under-expressed in cancer (33).

One of the most important features of miRNAs is that they have different expression patterns in normal cells when compared with cancer cells, which makes them excellent candidates for biomarkers (37, 38). In addition, miRNA expression signatures in blood, serum and plasma are similar between species, as well in individuals of different ages from the same species (38). Specific expression patterns of serum miRNAs have already been identified for pregnancy, diabetes, and different cancers, thus providing evidence that plasma miRNAs contain fingerprints distinctive of certain human conditions (19). Circulating miRNAs are also stable after being submitted to severe conditions such as boiling, very low or high pH, extended storage, and several freeze-thaw cycles, that would normally degrade most RNAs (19). They also seem to be protected from RNase activity, which solves the problem of possible degradation and launches them as one of the top candidates for circulating biomarkers.

## Circulating tumor-microvesicles as potential microRNA carriers

Over the past decade, tumors have increasingly been recognized as organs whose complexity approaches and may even exceed that of normal healthy tissues. When analyzed from this point of view, the biology of a tumor can only be understood by studying the individual specialized cell types within it as well as the “tumor microenvironment” that it is assembled during the course of tumorigenesis. This approach contrasts with the earlier view of a tumor as nothing more than a cluster of transformed cells standing alone, whose entire biology could be understood by elucidating their cell-autonomous properties (39).

Cancer cells in primary tumors are surrounded by a complex microenvironment. This microenvironment is composed by numerous types of cells including endothelial cells of the blood and lymphatic circulation, stromal fibroblasts and a variety of bone-marrow-derived cells including macrophages, myeloid-derived suppressor cells, TIE-2 expressing monocytes and mesenchymal stem cells (40).

**Figure 2.**
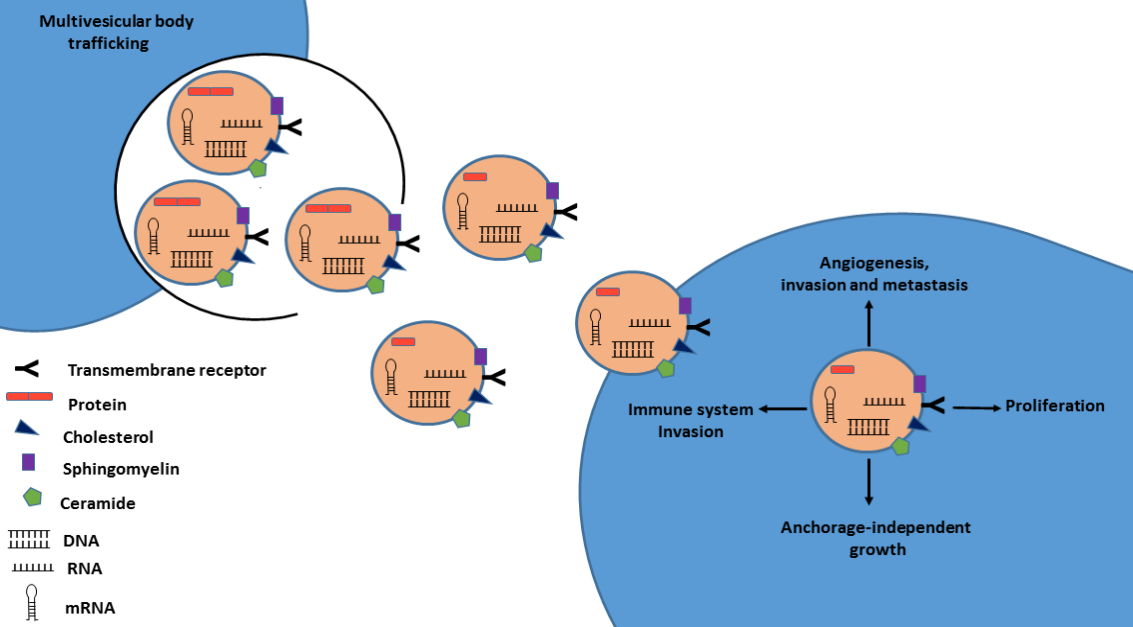
Schematic model of exosome secretion in cancer cells. Exosome membranes are enriched in cholesterol, sphingomyelin, and ceramide, as well as lipid raft associated proteins. These components allow exosomes to be highly stable in numerous body fluids. Exosomes released from cancer cells transport a variety of molecules (miRNAs, RNAs, DNA, proteins) and can be taken up by neighboring cells and are capable of inducing pathways involved in cancer initiation and progression.

One way of microenvironment modulation is through paracrine and/or systemic signaling between cells. This type of intercellular communication can occur by a direct cell-to-cell contact through adhesion junctions or by releasing of soluble signaling molecules (growth factor, cytokines) by the exchange of cellular fragments such as extracellular membrane microvesicles (EMV) (41). Shed EMVs serve to shuttle bioactive molecules between cells and their cargo can modulate the extracellular microenvironment (42). The EMVs are small circulating fragments (40-5000 nm diameter) with characteristics of the cell origin, that can be categorized into exosomes, microvesicles or ectosomes, apoptotic bodies or Golgi vesicles based on their size, origin, morphology and mode of release (38, 43). The best characterized EMVs are the exosomes, 50- to 100-nm vesicles generated intracellular in multi-vesicular bodies (MVBs) and released directly or upon fusion with the plasma membrane (Figure 2) (42, 44). The EMVs are bilayered membrane vesicles secreted by all cell types that can be released in the interstitial space or into circulating body fluids, which allows the travelling of these vesicles and the posterior binding and entrance in receptors cells (41).

Exosome-mediated cell communication includes, but is not restricted to, direct activation of cell-surface receptors on recipient cells, transfer and translation of mRNAs, transfer of miRNAs and silencing of mRNA targets, transfer of functional proteins and the induction of cell signaling pathways upon their internalization (42, 45). Valadi and co-workers were the first group to observe the existence of miRNAs in exosomes, which can be delivered to another cell, and remain functional in the receptor cells (46).

A recent study performed by King and co-workers provides evidence that microenvironment modulation (oxygen oscillations) promotes exosome release from breast cancer cells, which could be mediated by HIF-1α signaling pathway (47). This scenario is particularly important in RCC where VHL inactivation leads to the accumulation of HIF-1α and the activation of HIF-regulated molecules (2). Moreover, Kahlert and co-workers found that exosomes from serum of pancreatic cancer patients can be used for genomic DNA mutations detection, with impact on cancer prediction and treatment (48). Thus, exosomes can select bioactive molecules and propagate the horizontal transfer of their cargo and, consequently, have an enormous impact on tumor growth, angiogenesis, escape from immune surveillance, extracellular matrix degradation and metastasis (44, 49, 50). Since they are released into the circulation, exosome-dependent signaling may occur not only locally, but also in a paracrine and systemic manner, which can have a direct impact in tumor progression and metastasis (45).

The elevated stability of miRNA in circulating samples is thought to be the result of their capacity to circulate inside of exosomes (42). Exosome membranes are enriched in cholesterol, sphingomyelin, and ceramide as well as lipid raft associated proteins (51, 52). These components allow exosomes to be highly stable and thus be collected from numerous body fluids including blood, urine, breast milk, ascites and saliva (^53-58^). The lipid content of membranes is important because cholesterol depletion results in the inhibition of EMV release (42, 59). Thus, we hypothesize that higher levels of cholesterol observed in obese individuals can lead to an increase of EMV formation and release which in turn could promote miRNAs networks disruption leading to cardiovascular diseases and cancer development.

Differential expression of miRNA between normal and cancer patients has been reported (60–62). Although normal cells within the peripheral circulation can contribute to exosome population, the primary source of circulating exosomes in cancer patients is the tumor (63). Several reports indicate that cancer cells release more extracellular vesicle than normal cells and that the biomolecular cargo (nucleic acids, proteins and lipids) is reflective of the cell of origin (43). Moreover, miRNA containing tumor-derived exosomes can affect biological processes inside of recipient cells and, consequently, affect the tumor microenvironment (42). MiRNA molecules have also been described in exosomes shed from several tumor cell lines, including lung, glioblastoma and gastric cancers (60, 61, 64). It has also been suggested that tumor derived exosomes could be vehicles involved in the metastization process. Grange and co-workers found that CD105-positive exosomes (containing miRNAs) that were released by renal cell carcinoma stem cells triggered angiogenesis and the formation of a pre-metastatic niche in the lungs, when injected in mice (65). Circulating extracellular vesicles derived from RCC contain miRNAs, such as miR-200c, miR-92, miR-141, miR-19b, miR-29a, miR29c, miR-650, and miR-151. These miRNAs have been associated with tumor invasion and metastasis (37, 65).

## Circulating microRNAs in Renal Cell Carcinoma

The majority of the efforts made with the purpose of finding a signature of deregulated miRNAs in RCC have used genome-wide microarray profiling on tissue samples (66, 67). Since microarray allows the analysis of hundreds of miRNAs at the same time, it is an easy way to get an overall view of differentially expressed miRNAs in RCC.

Several up-regulated miRNAs have been described in RCC tissue samples, such as the miR-210 and miR-155, whose expressions can be induced by the hypoxic tumor microenvironment of RCC (68, 69). Furthermore, the down-regulation of miR-141, miR-149 and miR-200c are also described in these tumors. The down-regulation or loss of these miRNAs is associated with epithelial–mesenchymal transition (EMT) (38). MiR-200c can target the ZEB1, a transcription factor that drives the EMT process (70). Youssef and coworkers developed a ‘decision tree’ based on miRNA expression signature of RCC samples (71). The ‘decision tree’ can enable researchers to distinguish different subtypes of RCC. The system has a sensitivity of 97% in distinguishing normal from RCC, 100% for clear cell RCC subtype, 97% for papillary RCC subtype and 100% accuracy in distinguishing oncocytoma from chromophobe RCC subtype (71). While these results are promising, they were generated using a limited number of samples. Furthermore, obtaining tissue samples require the use of invasive biopsy (66).

Hence, profiling miRNA signature in biofluids is attractive strategy. In this regard, only a few studies have assessed circulating miRNA in RCC as potential biomarkers (Table 1).

**Table 1.** Summary of circulating miRNAs detected in RCC patients

MIRNA	POPULATION (SIZE)	TYPE OF SAMPLE	MIRNA REGULATION	CLINICAL IMPLICATIONS	REF
MIR-15A	7 RCC/ 5 chRCC*/6 pRCC/ 5 Onco/ 5 HC	urine	Up	diagnosis of ccRCC	(81)
MIR-210	34 RCC/ 23 HC	serum	Up	diagnosis of RCC	(77)
	68 RCC/ 42 HC	serum	Up	diagnosis of RCC	(76)
MIR-221	43 RCC / 34 HC	plasma	Up	diagnosis and prognosis of RCC	(72)
MIR-378	90 RCC/ 35 HC	serum	Up	diagnosis of RCC	(27)
MIR-451	90 RCC/ 35 HC	serum	Down	diagnosis of RCC	(27)
MIR-508-3P	10 RCC/ 10 HC	serum	Down	diagnosis of RCC	(80)
MIR-1233	84 RCC/ 93 HC	serum	Up	diagnosis of RCC	(26)

* RCC, renal cell carcinoma; chRCC, chromophobe RCC; pRCC, papillary RCC; Onco, oncocytoma; HC, healthy controls.

Teixeira and co-workers suggested that plasma level of miR-221 plasma is a potential biomarker of RCC progression. By integrating histopathological characteristics, patients’ age and miR-221 plasma expression levels, the authors proposed that higher circulating expression levels of miR-221 associated with poor overall survival (72). Furthermore, patients with metastatic RCC on diagnosis presented 10.9-fold increase of miR-221 expression when compared to patients with localized disease (72). The miR-221 was identified as a downstream target of EGFR-RAS-RAF-MEK pathway, and the down-regulation of this miRNA is associated with the inhibition of the invasion potential and the secretion of matrix metalloproteinase 2 and 9 (73-75).

The serum levels of miR-210 were proposed as biomarkers for molecular diagnosis of ccRCC by Zhao and coworkers (76). A decrease in serum miR-210 levels was observed after nephrectomy, emphasizing the hypothesis of miRNA release from tumor into circulation (76). Iwamoto and co-workers also observed a higher expression of miR-210 in tumor versus normal tissue samples from 34 RCC patients. However, no statistically significant association was found when matched for age, gender, tumor size or metastases (77). The expression of miR-210 is directly regulated by hypoxia and has the potential to be a biomarker of HIF-α pathway activation. Furthermore, miR-210 has multiple direct targets and exerts its influence on a wide range of cellular processes such as proliferation, differentiation, mitochondrial metabolism, protein modification, nucleic acid binding, migration and angiogenesis (78).

Redova and coworkers showed that miR-451 is down-regulated in serum samples of RCC patients when compared to healthy individuals. They also demonstrated that miR-378, which is known to promote cell survival and angiogenesis, is up-regulated in serum samples of RCC patients (n=90) (27). However, in the same year Hauser and co-workers, using serum samples of 117 RCC patients, did not observe this up- regulation compared with the levels observed in healthy individuals. Moreover, these authors did not find any statistically significant association between the expression of miR-378 and pT-stage, lymph node/distant metastasis, vascular invasion and Fuhrman grade (79). The contradictions observed between the two studies could be the consequence of differences in the biologic populations (patients and control group) and also the influence of the methodology used for miRNA extraction/purification.

Zhai and co-workers observed a down-regulation of miR-508-3p in biopsy samples of RCC patients and validated the results in 10 plasma samples of the same RCC patients (80). They also proposed that miR-508-3p would play an important role as tumor suppressor gene during tumor formation and that it may serve as novel diagnostic marker for RCC (80).

The high serum levels of miR-1233 were described by Wulfken and co-workers in RCC patients (n=84 patients) compared with healthy controls, with a sensitivity of 77.4% and a specificity of 37.6% (26). Using a bioinformatics approach (miRWalk) the authors suggested that this miRNA can target p53 and BLCAP, well-known tumor suppressor genes (26).

Another biological sample with a potential to be used for molecular biomarkers detection is the urine. Von Brandenstein and co-workers proposed that miR-15a may be an important biomarker aiding in ccRCC detection since it is detectable in the urine of ccRCC patients but is nearly undetectable in the urine of patients with other urinary tumors, and urinary tract inflammation (81). Recently, Komabavashi and co-workers proposed that the down regulation of miR-15a is implicated in the pathogenesis of nasal NK/T-cell lymphoma, where it induces cell proliferation via MYB and cyclin D1 (82).

## Correlation between circulating microRNAs, VHL deregulation and renal cell carcinoma

The different RCC histological subtypes reflect differences in the molecular mechanisms involved in tumorigenesis as well as different prognosis. The papillary RCC is characterized by the gain of chromosome material (Trisomy 7, 17), chromophobe RCC is characterized by the loss of genetic material that included monosomy of chromosomes 1, 2, 6, 13, 17 or 21 (83), and the majority of cell RCC is characterized by loss of function of the von Hippel-Lindau (VHL) gene (84–87).

**Figure 3.**
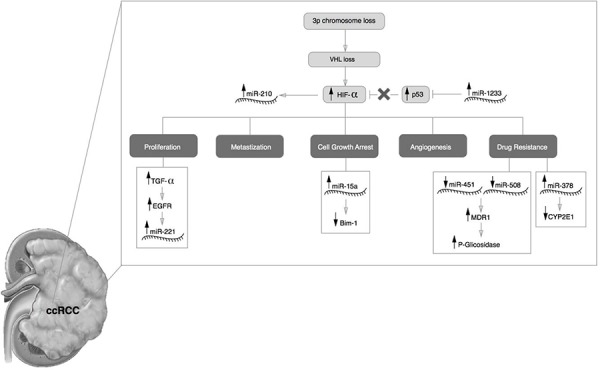
**VHL and miRNA**. Proposed mechanistic model for the role of miR-210, miR-1233, miR-221, miR-15a, miR-451, miR-508-3p, miR-378 during the ccRCC development.

In normoxic conditions, the protein encoded by the VHL gene (pVHL) serves as a recognition site for the regulatory subunits of HIF, targeting them for proteasomal degradation. One of the early molecular events of ccRCC is the loss of pVHL function (a consequence of the loss of the short arm of chromosome 3), which stops the degradation of HIF and leads to its accumulation in the cytoplasm and further migration to the nucleus where it binds to hypoxia-regulated genes. Once activated, these genes are involved in pathways responsible for angiogenesis, proliferation, glucose metabolism, pH regulation and metastatic disease (2, 87–89). Based on the previous section on circulating microRNAs in renal cell carcinoma (see above) we propose a possible mechanistic association between miRNAs and the VHL signaling pathway (Figure 3).

VHL deregulation induces the expression of miR-210 (90). MiR-1233, which is rapidly induced by hypoxia, in turn work together with HIF-α to regulate the hypoxic response from the cell (91, 92). Thus, we hypothesize that miR-210 is induced by hypoxia and miR-1233 helps to maintain the hypoxic status.

The VHL is also responsible for EGFR (epidermal growth factor receptor) turnover. Studies performed by Zhou and co-workers showed that the half-life of EGFR is longer in cells lacking the VHL gene compared to normal cells (90). The EGFR stabilization in membrane and the TGFα biodisponibility (as a consequence of VHL deregulation) can promote the constant activation of EGFR-RAS-RAF-MEK leading to a higher cellular proliferation. Since the miR-221 is induced by EGFR-RAS-RAF-MEK pathway, it is fitting that higher levels of this miRNA is found in patients with ccRCC (72). MiR-221 can actively repress TIMP-3 and cell cycle inhibitor proteins p27/Kip1 and p57, facilitating cell proliferation, self-renewal and epithelial-mesenchymal transition of RCC (93, 94).

Recently, Camps and co-workers observed an increase of miR-378 at lower concentrations of oxygen, suggesting that this miRNA could be a potential biomarker for hypoxia (95). It is known that miR-378 inhibits the expression of CYP2E1, which could be implicated in chemotherapy responses (96).

Kozakowski and co-workers found a higher expression of Bmi-1 (B lymphoma mouse Moloney leukemia virus insertion region) during RCC development. Bmi-1 is indispensable for the self-renewal of neural and hematopoietic stem cells, and a high expression is observed in papillary RCC and oncocytomas. However, in ccRCC, Bmi-1 expression was inversely correlated (97). Bim-1 down-regulation in ccRCC could be explained by the capacity of miR-15a to target the Bmi-1 3’ UTR mRNA leading to it degradation or translational repression (98).

Other circulating miRNAs deregulated in RCC were the miR-451 and miR-508-3p (80, 99). MiRNA-451 and miR-508-3p are involved in the regulation of MDR1 gene (multidrug resistance 1 gene) that encode the human P-glycoprotein (100, 101). The elevated levels of P-glycoprotein in cytoplasm and membranes are associated with drug-resistance of tumors (100). The significant role of P-glycoprotein in drug pharmacokinetics is suggested by its location in the adrenal gland and in proximal tubules of the kidney (102). MiR-451 reduces the expression of MDR1 mRNA and P-glycoprotein. A reduction of miR-451 and miR-508-3p could be reason why RCC is notoriously resistant to conventional therapeutics.

## Conclusion

The stability and “anti-degradation” nature of exosomes in body fluids and the variety of molecules that they carry, such as miRNAs, makes them an ideal target for biomarkers discovery since their cargo reflects the characteristics of the cell of origin. Nevertheless, the knowledge regarding the mechanisms in which miRNAs are selected and incorporated in exosomes is limited and further investigations is needed to clarify the biological impact of these molecules in distant sites of the body. In the future, clarification of these mechanisms will enable the elucidation of the metastatic process and the discovery of new cancer therapies. Several studies were able to detect circulating miRNAs in body fluids of RCC patients, supporting their suitability as biomarkers. The use of biological fluids such as plasma, serum and urine may open the door to the so called “liquid biopsies”, a less invasive method that could effectively overcome the challenges associated to conventional tissue sampling and provide more sensitive biomarkers. However, the establishment of standard protocols for isolation and quantification of miRNAs are needed in order to implement their use as biomarkers in the clinical practice.
